# Declining research interest among oncology residents: insights into the academic career from a nationwide longitudinal study in France

**DOI:** 10.1016/j.esmoop.2025.106025

**Published:** 2026-01-02

**Authors:** N. Taranto, M. Duval, C. Raynaud, A. Boilève, N. Naoun, A. Rousseau, E. Ashton, L. Ollivier, J. Alexandre, F. Huguet, M. Kfoury, M. Delaye, M. Hilmi

**Affiliations:** 1 Medical Oncology Department, Curie Institute, Saint Cloud, France; 2 Association for the Education and Research of Oncology Residents (AERIO), Paris, France; 3 French Society of Young Radiation Oncologists (SFjRO), Paris, France; 4 Medical Oncology Department, Gustave Roussy, Villejuif, France; 5 Medical Oncology Department, Georges Pompidou European Hospital, Paris, France; 6 Department of Radiation Oncology, Institut de Cancérologie de l’Ouest – Centre René Gauducheau, Nantes, France; 7 Medical Oncology Department, Cochin Hospital, Paris, France; 8 Department of Radiation Oncology, Tenon Hospital, AP-HP, Sorbonne University, Paris, France; 9 Medical Oncology Department, Paoli-Calmettes Institute, Marseille, France

**Keywords:** oncology workforce, medical education, academic career, career aspirations, clinician-scientist

## Abstract

**Background:**

Oncology demands both high-quality clinical care and continuous research innovation. Yet, sustaining an academic workforce remains a challenge. Although prior studies have explored residents' motivations, longitudinal insights into their evolving academic interests are lacking.

**Materials and methods:**

We conducted a nationwide, longitudinal survey of French oncology residents (2020-2023), targeting first-year cohorts annually. The study included residents from both medical oncology and radiation oncology. The 42-item questionnaire assessed academic aspirations, research involvement, clinical workload, and career priorities.

**Results:**

Among 498 eligible residents, 377 responded (75.7%). Over time, interest in research declined (79.4% in 2020 versus 63.3% in 2023, *P* < 0.05), as did intentions to pursue Master of Science (MSc) (32.3% versus 17.4%) and Doctor of Philosophy (PhD) programs (17.6% versus 7.3%). Notably, access to supervised research time improved (22.6% to 37.4%, *P* < 0.05), yet scientific society memberships fell (43.5% to 25.2%). Of residents responding more than once (*n* = 54), 44.4% abandoned PhD aspirations. Key barriers included lack of time (65.1%), mentorship (44.1%), and financial support (31.6%).

**Conclusion:**

Our findings reveal a worrying decline in academic engagement among French oncology residents, echoing global trends. Addressing this requires structured mentorship, protected research time, and flexible academic pathways to sustain the clinician-scientist pipeline.

## Highlights

• Longitudinal survey of 377 French oncology residents (2020-2023).
• Research motivation declined from 79% to 63% over 4 years.
• MSc and PhD interest dropped by half during residency training.
• Mentorship, time, and funding identified as main barriers.

## Introduction

The global incidence of cancer is steadily rising, increasing the demand for specialized oncologic care and research. According to the World Health Organization, cancer remains a leading cause of death worldwide, a trend expected to continue in the coming decades.^1^ This underscores the urgent need for a well-trained oncology workforce and stronger research efforts.

Training future oncologists, both clinicians and clinician-scientists, is vital for sustaining high-quality care and driving scientific innovation. After residency, career options span private practice, general hospitals, university hospitals, and comprehensive cancer centers. Academic careers require additional Master of Science (MSc) and Doctor of Philosophy (PhD) training, which is often pursued in university or cancer centers with the necessary infrastructure.^2^

In France, oncology training consists of 6 years of medical school followed by a 5-year residency across 49 oncology training centers, distributed evenly across the country according to regional population density.^3^ Residency positions are allocated through a national written examination based solely on medical knowledge, with rankings determining placement; prior research experience is not considered. During residency, trainees rotate through different hospital departments every 6 months. They choose between medical oncology and radiation oncology at the end of their second year. Upon completion of residency, trainees must defend a medical thesis based on an original research project. Residents’ associations may provide financial support for research-related activities such as conference attendance or scientific society membership. Additional theoretical specialization can be pursued through optional complementary certifications. Clinical subspecialization is not required during residency but typically occurs afterward through a mandatory clinical fellowship, whereas research fellowships are less common and generally undertaken only after obtaining an MSc or PhD.

Previous studies show many French residents are interested in research, but face barriers such as time constraints, lack of mentorship, and limited financial incentives.^4^ Furthermore, there is widespread concern about the difficulty of securing academic positions after residency and increasing competition for such roles.^5^

Building on these findings, our study uses a longitudinal approach to examine how the academic interests and career aspirations of oncology residents evolve over time. This design offers a comprehensive view of shifting motivations and challenges throughout their training.

## Materials and methods

We conducted a national, descriptive, longitudinal, and anonymous online survey across first-year oncology residents from December 2020 to December 2023. Residents were contacted by national oncology residents’ associations (AERIO: Association for the Education and Research of Oncology Residents; SFjRO: French Society of Young Radiation Oncologists), that were responsible for diffusing the questionnaire via their e-mail distribution lists, websites, and social networks.

All residents entering their first year between December 2020 and December 2023 were invited to participate at the start of their residency and then followed up with reminders every year throughout their training, until April 2024. Therefore, fifth-year residents were never surveyed.

The survey included 42 questions across six sections: demographics (7 questions), academic dimension (11 questions), clinical dimension (9 questions), training (9 questions), life and career goals (4 questions), and associative involvement (2 questions) (Supplementary Table S1, available at https://doi.org/10.1016/j.esmoop.2025.106025). All potential answer choices for non-ranking questions are reported in the results tables. For ranking questions, respondents were asked to rank the proposed options from 1 (most desired) to *n* (least desired) depending on the number of possible choices, and the mean ranking score for each category is presented in the results tables. The questions were selected by the resident associations (AERIO and SFjRO) in collaboration with oncology professors. The same questionnaire was previously used and published in our first study on first-year residents.^5^ In the present work, we used this same instrument to assess responses over time. We included all responses provided by participants, even if the questionnaire was not fully completed. The first part of our study focused on analyzing the responses provided by first-year residents over time. The second part of our study focused on residents who responded at least twice during their training, with the second response occurring from their second year onward. We then compared each resident’s first and last responses. The survey was hosted on sondageonline.fr.

Continuous variables were summarized as medians with interquartile ranges (IQR) and compared across cohorts using the Kruskal–Wallis test or one-way analysis of variance when appropriate. Categorical variables were compared using the chi-square or Fisher’s exact test. All reported *P* values were two-sided, with statistical significance defined as *P* < 0.05. The cluster analysis was conducted to identify distinct motivational and academic profiles among residents. Responses to categorical survey items related to research motivation and engagement were converted into binary variables using one-hot encoding. Mixed data were analyzed using Gower distance, computed with the daisy() function from the cluster package. Hierarchical clustering was then carried out with the Ward.D2 linkage method. All analyses were carried out using R (version 4.2.3).

## Results

### Demographics

Out of 498 first-year residents invited to participate (119 in 2020, 121 in 2021, 126 in 2022, and 132 in 2023), 377 responded to the survey (72 in 2020, 95 in 2021, 101 in 2022, and 109 in 2023), representing an average response rate of 75.7% (Supplementary Table S2, available at https://doi.org/10.1016/j.esmoop.2025.106025). Ninety-six percent of respondents completed the entire questionnaire. Demographic characteristics were consistent across years (*P* > 0.05): the majority were female (58.1%), single (62.1%), with a median age of 24 years (IQR 24-25 years). Oncology was the first-choice specialty for nearly all residents regardless of cohort year (91.5%). Upon entering residency, most residents were undecided between medical and radiation oncology (88.3%).

### Clinical dimension

Most residents reported working 45-55 hours per week (55.4%), with 2-3 on-call shifts per month (55.9%) and <4 on-call duty weekends per semester (74.5%) (Table 1). Nearly half of the residents had not yet chosen a subspecialty (51.3%). Among those with preferences, the most popular were breast oncology (33.6%), gynecologic oncology (22.8%), gastrointestinal oncology (20.7%), and thoracic oncology (19.1%). A total of 63.7% of residents expressed interest in pursuing a complementary certification during residency. The most considered were palliative care (30.1%), pain management (20.2%), pediatric oncology (18.0%), genetics and molecular medicine (13.7%), pharmacology (7.3%), and medical bioinformatics (5.4%).

**Table 1:** Evaluation of the clinical perspective

		2020 (*n* = 69), *n* (%)	2021 (*n* = 94), *n* (%)	2022 (*n* = 100, *n* (%))	2023 (*n* = 109), *n* (%)	*P*
**Average weekly hospital work, hours**	**<45**	10 (14.5)	14 (14.9)	23 (22.0)	21 (19.2)	0.81
	**45-55**	40 (58.0)	54 (57.4)	53 (53.0)	59 (54.1)	
	**>55**	19 (27.5)	26 (27.7)	24 (24.0)	29 (26.7)	
**Average number of on-call shifts per month**	**0-1**	27 (39.1)	31 (33.0)	40 (40.0)	51 (46.8)	0.33
	**2-3**	37 (53.6)	59 (62.8)	56 (46.0)	56 (51.4)	
	**4 or more**	5 (7.2)	4 (4.3)	4 (4.0)	2 (1.8)	
**Average number of on-call weekends per semester**	**0**	32 (46.4)	45 (49.7)	43 (43.0)	61 (56.0)	0.26
	**1-3**	17 (24.6)	21 (22.3)	28 (28.0)	30 (27.5)	
	**4-6**	12 (17.4)	22 (23.4)	20 (20.0)	15 (13.8)	
	**>6**	8 (7.3)	6 (6.4)	9 (9.0)	3 (2.7)	
**Desire to pursue a complementary certification**^a^	**No**	4 (5.8)	12 (12.8)	14 (14.0)	15 (13.8)	0.55
	**Maybe**	46 (66.7)	55 (58.5)	64 (64.0)	72 (66.1)	
	**Pharmacology**	10 (14.5)	6 (6.4)	5 (5.0)	6 (5.5)	
	**Pain management**	12 (17.4)	20 (21.3)	26 (26.0)	17 (15.6)	
	**Palliative care**	23 (33.3)	30 (31.9)	31 (31.0)	28 (25.7)	
	**Medical bioinformatics**	5 (7.2)	6 (6.4)	5 (5.0)	4 (3.7)	
	**Medical expertise and personal injury**	3 (4.3)	1 (1.1)	3 (3.0)	3 (2.8)	
	**Genetics and molecular medicine**	12 (17.4)	15 (16.0)	13 (13.0)	11 (10.1)	
	**Oncology (hematology and pediatrics)**	15 (21.7)	20 (21.3)	10 (10.0)	22 (20.2)	
**Desired subspecialty in oncology**^a^	**General oncology**	20 (29.0)	29 (30.9)	19 (19.0)	36 (33.0)	0.74
	**Gastrointestinal oncology**	13 (18.8)	21 (22.3)	19 (19.0)	24 (22.0)	
	**Gynecological oncology**	14 (20.3)	24 (25.5)	14 (14.0)	33 (30.3)	
	**Breast oncology**	18 (26.1)	33 (35.1)	32 (32.0)	42 (38.5)	
	**Urological oncology**	12 (17.4)	16 (17.0)	11 (11.0)	19 (17.4)	
	**Neurological oncology**	13 (18.8)	14 (14.9)	9 (9.0)	19 (17.4)	
	**Ear, nose, throat oncology**	4 (5.8)	8 (8.5)	6 (6.0)	6 (5.5)	
	**Thoracic oncology**	12 (17.4)	15 (16.0)	23 (23.0)	21 (19.3)	
	**Dermatological oncology**	6 (8.7)	12 (12.8)	10 (10.0)	18 (16.5)	
	**Rare tumors oncology**	9 (13.0)	15 (16.0)	13 (13.0)	18 (16.5)	
	**Unknown**	38 (55.1)	41 (43.6)	62 (62.0)	50 (45.9)	
**Desired internship areas for free semesters**^a^	**Palliative care**	24 (34.8)	36 (38.3)	43 (43.0)	42 (38.5)	0.83
	**Organ specialty**	32 (46.4)	40 (42.6)	42 (42.0)	43 (39.4)	
	**Pathology**	15 (21.7)	19 (20.2)	14 (14.0)	21 (19.3)	
	**Imaging**	41 (59.4)	46 (48.9)	47 (47.0)	55 (50.5)	
	**Genetics, molecular biology**	10 (14.5)	13 (13.8)	9 (9.0)	9 (8.3)	
	**Public health**	2 (2.9)	3 (3.2)	3 (3.0)	3 (2.8)	
	**Pharmacology**	5 (7.2)	1 (1.1)	2 (2.0)	0 (0)	
	**Intensive care**	35 (50.7)	53 (56.4)	48 (48.0)	63 (57.8)	
	**Internal medicine**	23 (33.3)	36 (38.3)	31 (31.0)	27 (24.8)	
	**Other**	7 (10.1)	12 (12.8)	14 (14.0)	20 (18.3)	
**Motivation to pursue oncology**^a^	**Financial compensation**	23 (33.3)	38 (40.4)	40 (40.0)	46 (42.2)	0.90
	**Moderate workload**	11 (15.9)	24 (25.5)	26 (26.0)	33 (30.3)	
	**Preferred human contact**	64 (92.8)	85 (90.4)	93 (93.0)	100 (91.7)	
	**Innovative specialty**	64 (92.8)	85 (90.4)	92 (92.0)	91 (83.5)	
	**Scientific interest**	58 (85.1)	74 (78.7)	74 (74.0)	78 (71.6)	
	**Personal experience (close relative affected)**	19 (27.5)	26 (27.7)	25 (25.0)	33 (30.3)	
	**Other internship as a student**	43 (62.3)	46 (48.9)	67 (67.0)	54 (49.5)	
	**Transversality and multidisciplinary work**	63 (91.3)	87 (92.6)	88 (88.0)	95 (87.2)	
	**International mobility at conferences**	17 (24.6)	29 (30.9)	22 (22.0)	38 (34.9)	
	**Technical procedures**	8 (11.6)	10 (10.6)	14 (14.0)	22 (20.2)	
	**Prestige of the specialty**	11 (15.9)	23 (24.5)	19 (19.0)	27 (24.8)	
**Concerns about oncology**^a^	**Heavy workload**	32 (46.4)	50 (53.2)	62 (62.0)	64 (58.7)	0.85
	**Low compensation**	8 (11.6)	9 (9.6)	9 (9.0)	9 (8.3)	
	**Conflicts of interest**	1 (1.4)	3 (3.2)	6 (6.0)	6 (5.5)	
	**Contact with death**	32 (46.4)	40 (42.6)	45 (45.0)	49 (45.0)	
	**Fear of career opportunities**	18 (26.0)	18 (19.1)	24 (24.0)	20 (18.3)	
	**Management of medical situations**	16 (23.2)	30 (31.9)	26 (26.0)	33 (30.3)	
	**Multidisciplinary work**	4 (5.8)	0 (0.0)	6 (6.0)	2 (1.8)	
	**Over-specialization**	26 (37.7)	42 (44.7)	40 (40.0)	40 (36.7)	
	**Competition with organ specialists**	24 (34.8)	34 (36.2)	34 (34.0)	28 (25.7)	
	**Lack of diagnoses**	14 (20.3)	23 (24.5)	31 (31.0)	22 (20.2)	
	**None**	5 (7.2)	5 (5.3)	5 (5.0)	10 (9.2)	
**Has considered changing specialty**		10 (14.5)	16 (17.0)	13 (13.0)	15 (13.8)	0.87
**If yes, expressed reasons**^a^	**Heavy workload**	3 (30.0)	11 (68.8)	5 (38.5)	7 (50.0)	0.70
	**Low compensation**	0 (0.0)	0 (0.0)	1 (7.7)	1 (6.7)	
	**Conflicts of interest**	0 (0.0)	0 (0.0)	0 (0.0)	0 (0.0)	
	**Contact with death**	5 (50.0)	6 (37.5)	3 (23.1)	4 (26.7)	
	**Fear of career opportunities**	4 (40.0)	3 (18.8)	4 (30.8)	0 (0.0)	
	**Management of medical situations**	0 (0.0)	3 (18.8)	5 (38.5)	2 (13.3)	
	**Multidisciplinary work**	0 (0.0)	0 (0.0)	0 (0.0)	0 (0.0)	
	**Over-specialization**	1 (10.0)	3 (18.8)	4 (30.8)	3 (20.0)	
	**Competition with organ specialists**	1 (10.0)	2 (12.5)	3 (23.1)	1 (6.7)	
	**Other**	3 (30.0)	4 (25.0)	4 (30.8)	3 (20.0)	

a Multiple responses allowed.

Motivations for entering oncology were consistent across cohorts, with top factors being valued human contact (91.9%), the interdisciplinary nature of care (89.5%), and the innovative character of the specialty (89.2%). The moderate workload was increasingly cited, rising from 15.9% in 2020 to 30.3% in 2023 (*P* = 0.03). Main concerns included significant workload (55.9%), contact with death (44.6%), and over-specialization (39.8%). Fear of low remuneration was noted by 9.4% of residents. Regarding specialty change, 14.5% had considered it early on, primarily due to excessive workload (48.1%), contact with death (33.3%), career uncertainty (20.4%), complexity of medical situations (18.5%), over-specialization (20.4%), and competition with organ specialists (13.0%).

### Interest in research

**Figure 1:**
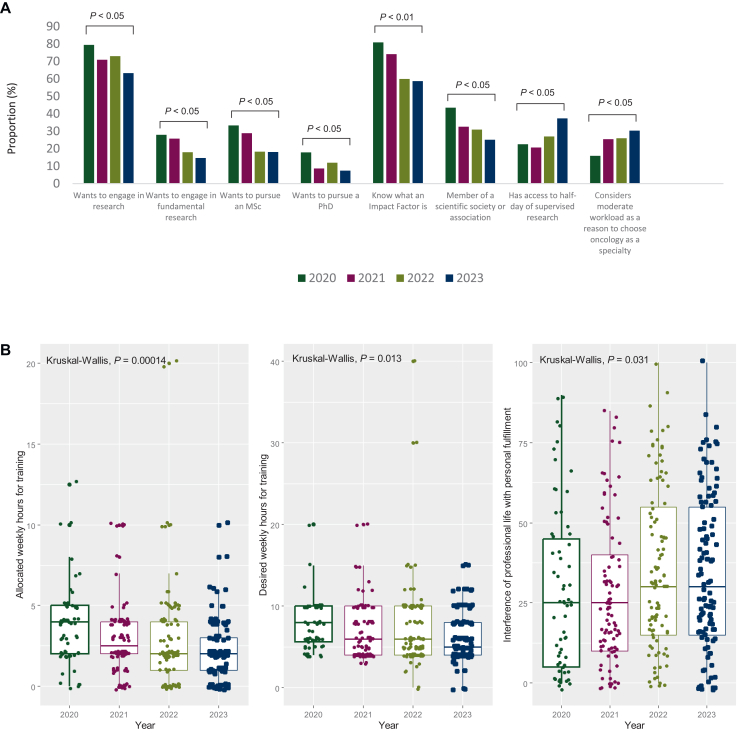
**Change over time in research aspirations and workload perception among first-year oncology residents.** (A) Bar plots illustrating the percentage of oncology residents expressing interest in research, fundamental research, pursuing a Master of Science (MSc), or a Doctor of Philosophy (PhD), as well as their familiarity with the concept of an Impact Factor. Additionally, it shows membership in scientific societies, access to supervised research time, and the perception of moderate workload as a motivating factor for choosing oncology. Data are presented for four consecutive years (2020 to 2023), represented by different colors.(B) Box plots depicting the shift in training hours and work-life balance changes over time (2020 to 2023).

Basic or fundamental research focuses on understanding biological mechanisms, often in the laboratory, without immediate clinical application. Translational research aims to apply these fundamental discoveries to develop new strategies for prevention, diagnosis, or treatment in the clinical setting. This section assessed the inclination of young oncology residents to engage in research, their motivations, barriers, and how these evolved (Figure 1A). In 2020, 20.6% did not wish to do research; this rose steadily to 36.7% in 2023 (*P* = 0.02) (Table 2). Among those interested, clinical research was most appealing (67.3%), followed by fundamental (20.8%), and translational research (11.9%). Interest in fundamental research declined from 29.7% in 2020 to 14.7% in 2023 (*P* = 0.03). Awareness of the concept of impact factor dropped, with 19.1% unfamiliar in 2020 versus 41.3% in 2023 (*P* < 0.01). Understanding of translational research remained low (18.1%).

**Table 2:** Evaluation of the interest in research

		2020 (*n* = 68), *n* (%)	2021 (*n* = 93), *n* (%)	2022 (*n* = 100), *n* (%)	2023 (*n* = 109), *n* (%)	*P*
**Knows what translational research is**		8 (11.8)	17 (18.3)	20 (20.0)	22 (20.2)	0.49
**Desired type of research**^a^	**None**	14 (20.6)	27 (29.0)	27 (27.0)	40 (36.7)	0.21
	**Clinical research**	46 (67.6)	64 (68.8)	71 (71.0)	68 (62.4)	
	**Translational research**	8 (11.8)	7 (7.5)	14 (14.0)	15 (13.8)	
	**Fundamental research**	19 (27.9)	24 (25.8)	18 (18.0)	16 (14.7)	
**Who motivated the resident to pursue research**^a^	**Nobody**	11 (16.1)	20 (21.5)	18 (18.0)	23 (21.1)	0.01
	**Faculty or non-faculty physician**	20 (29.4)	13 (14.0)	16 (16.0)	9 (8.3)	
	**Researcher**	12 (17.6)	8 (8.6)	7 (7.0)	6 (5.5)	
	**Other**	3 (4.4)	16 (17.3)	8 (8.0)	6 (5.5)	
**Desire to pursue an MSc**	**Yes**	22 (32.3)	26 (28.0)	17 (17.0)	19 (17.4)	0.25
	**Maybe**	33 (48.5)	47 (50.5)	51 (51.0)	58 (53.2)	
	**No**	11 (16.2)	17 (18.3)	25 (25.0)	28 (25.7)	
	**Already done**	2 (2.9)	3 (3.2)	7 (7.0)	4 (3.7)	
**Desire to pursue a PhD**	**Yes**	12 (17.6)	8 (8.6)	12 (12.0)	8 (7.3)	0.16
	**Maybe**	33 (48.5)	44 (47.3)	49 (49.0)	43 (39.4)	
	**No**	22 (32.3)	40 (43.0)	39 (39.0)	57 (52.3)	
	**Already done**	1 (1.5)	1 (1.1)	0 (0.0)	1 (0.9)	
**Desire to pursue a fellowship**	**Yes**	23 (33.8)	26 (28.0)	27 (27.0)	28 (25.7)	0.55
	**Maybe**	34 (50.0)	45 (48.4)	57 (57.0)	54 (49.5)	
	**No**	11 (16.2)	22 (23.7)	16 (16.0)	27 (24.8)	
**Motivations for research**^a^	**Science progress**	31 (45.6)	48 (51.6)	45 (45.0)	47 (43.1)	0.92
	**Diversity of practice**	41 (60.3)	51 (54.8)	47 (47.0)	43 (39.4)	
	**Access to an academic position**	14 (20.6)	17 (18.3)	20 (20.0)	19 (17.4)	
	**Personal pride**	12 (17.6)	28 (30.1)	25 (25.0)	16 (14.7)	
	**Intellectual stimulation**	45 (66.2)	63 (67.7)	65 (65.0)	65 (59.6)	
	**Traveling (conferences, mobility)**	10 (14.7)	21 (22.6)	23 (23.0)	18 (16.5)	
**Barriers to research**^a^	**Lack of information and guidance**	40 (58.8)	44 (47.3)	40 (40.0)	39 (35.8)	0.44
	**Lack of financial recognition**	27 (39.7)	24 (25.8)	35 (35.0)	31 (28.4)	
	**Lack of time**	43 (63.2)	68 (73.1)	61 (61.0)	69 (63.3)	
	**Lack of interest**	8 (11.8)	6 (6.5)	13 (13.0)	16 (14.7)	
	**Lack of self-confidence**	13 (19.1)	30 (32.3)	24 (24.0)	26 (23.9)	
	**Other**	1 (1.5)	3 (3.2)	1 (1.0)	3 (2.8)	
**Desire to publish in an international journal**	**Yes**	40 (58.8)	55 (59.1)	53 (53.0)	53 (48.6)	0.75
	**No**	27 (39.7)	42 (45.2)	50 (50.0)	55 (50.5)	
	**Already done**	2 (2.9)	1 (1.1)	2 (2.0)	2 (1.8)	
**Desire to present at national or international conferences**	**Yes**	33 (48.5)	43 (46.2)	42 (42.0)	49 (45.0)	0.99
	**No**	44 (64.7)	53 (57.0)	60 (60.0)	59 (54.1)	
	**Already done**	1 (1.5)	1 (1.1)	1 (1.0)	2 (1.8)	
**Knows what an impact factor is**	**Yes**	55 (80.9)	69 (74.2)	60 (60.0)	64 (58.7)	0.004
	**No**	13 (19.1)	24 (25.8)	40 (40.0)	45 (41.3)	

a Multiple responses allowed.

Motivation for doing research included intellectual stimulation (66.8%), diversity of practice (49.2%), and a desire to advance science (46.2%). Main barriers were lack of time (65.1%), insufficient guidance (44.1%), and limited financial support (31.6%). Interest in scientific communication remained steady: 54.3% wished to publish in an international journal, and 45.1% aimed to present at national or international conferences. However, interest in advanced academic degrees declined: MSc motivation fell from 32.3% in 2020 to 17.4% in 2023 (*P* = 0.02); PhD motivation declined from 17.6% to 7.3% (*P* = 0.03). Before starting oncology training, 4.3% and 0.8% reported holding an MSc or PhD, respectively. About one-third of residents (28.1%) considered a 1-year fellowship early in training.

**Figure 2:**
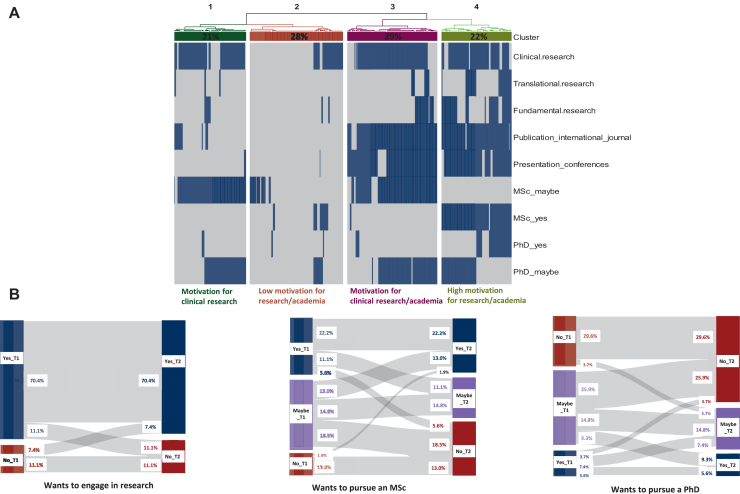
**Motivation of oncology residents to engage in research and academia.** (A) Heatmap visualizing the clustering of oncology residents based on their motivations and aspirations related to research and academia. The heatmap displays four distinct clusters, each corresponding to a unique profile of research motivation and academic aspirations. Blue cells indicate positive responses, and gray cells represent negative responses. Dendrograms at the top illustrate hierarchical clustering, visually separating the four distinct groups. The percentage labels above each cluster denote the proportion of residents belonging to each motivational profile. (B) Sankey diagrams depicting residents' motivations for engaging in research between their first (T1) and last (T2) response to the survey.

Figure 2A presents a cluster analysis of selected responses. Four clusters emerged, each reflecting different levels of motivation and involvement in research. Cluster 1 (21%) showed interest in clinical research but limited academic engagement. Cluster 2 (28%) reported low motivation and minimal participation in scientific activities. Cluster 3 (29%) expressed strong interest in both clinical and academic research, with more involvement in publications and conferences, and consideration of MSc or PhD programs. Cluster 4 (22%) was the most research-active, with substantial academic engagement and PhD plans. No significant associations were found between clusters and demographic or academic variables, including region of residency, gender, marital status, national ranking, or oncology discipline (all *P* > 0.10).

### Training

The median weekly time allocated to research by supervising physicians in hospitals decreased from 4 hours in 2020 (IQR 2-5 hours) to 2 hours in 2023 (IQR 1-3 hours) (*P* = 0.0001) (Supplementary Table S3, available at https://doi.org/10.1016/j.esmoop.2025.106025). Desired time for research also dropped, from 8 hours in 2020 (IQR 5.6-10 hours) to 5 hours in 2023 (IQR 4-8 hours) (*P* = 0.01) (Figure 1B). Access to a supervised research half-day increased significantly (22.6% in 2020 to 37.4% in 2023, *P* = 0.05), whereas access to a clinical or research training half-day remained stable (21.0% in 2020, 21.5% in 2023).

Most first-year residents (91.1%) wanted a mentor during training. Mentor choice was either indifferent (42.1%) or in favor of a clinical fellow (30.7%). Residents primarily sought guidance on career planning (83.1%), followed by teaching (71.2%), human aspects (53.7%), and research (52.1%).

Academic teaching received a moderate satisfaction score (53/100). Across all years, residents preferred hospital-based courses (mean ranking 2.11), followed by in-person university courses (2.52), e-learning (3.03), textbooks (3.18), and guideline articles (4.17).

Most residents preferred to engage with professional associations later in training (63.4%) (Supplementary Table S3, available at https://doi.org/10.1016/j.esmoop.2025.106025). However, scientific society membership declined from 43.5% in 2020 to 25.2% in 2023 (*P* = 0.01) (Figure 1A). Only 3.6% of first-year residents were European Society for Medical Oncology members.

### Life and career goals

Residents consistently ranked social and personal life as their top priority, followed by clinical work, financial aspects, and lastly, research (Supplementary Table S4, available at https://doi.org/10.1016/j.esmoop.2025.106025). The desire to have children decreased significantly, from 79.0% in 2020 to 63.6% in 2023 (*P* = 0.005). Perceived incompatibility between this goal and professional life increased from 3.2% to 8.4%. The perceived interference of professional life with personal fulfillment rose from a score of 25 to 30 (*P* = 0.03) (Figure 1B).

In terms of preferred future workplace, cancer treatment centers were consistently favored (mean rank 1.85), followed by private centers (2.58), public university hospitals (2.83), non-university public hospitals (3.13), industry (5.14), and research laboratories (5.56).

### Changes in responses throughout training

After comparing subsequent first-year cohorts, we aimed to evaluate individual-level changes over time, by analyzing answers from 54 residents (14.3%) who participated in the survey more than once, at least 6 months apart. Among them, 21 completed the survey for the last time during their second year, 22 during their third year, and 11 during their fourth year.

Among 44 residents initially motivated to do research (‘yes’ or ‘maybe’), 6 (13.6%) no longer wished to pursue it by their last response (Figure 2B). Of the 10 initially uninterested, 4 (40%) later expressed interest. For the MSc, 13 of 46 initially motivated residents (28.3%) eventually answered ‘no.’ Only one initially uninterested resident changed their mind. Overall, MSc interest declined significantly (85.2% to 63%, *P* < 0.01). Similarly, for the PhD, 16 of 36 initially motivated residents (44.4%) no longer wished to pursue it at their last response. None of the initially uninterested changed to ‘yes,’ confirming a significant drop in PhD interest (66.6% to 37.0%, *P* < 0.01). The proportion of residents completing an MSc during residency in France has been previously estimated at 29% in a study from our group,^6^ which aligns with the proportion of residents who remained motivated for research over time in our current study (36%, Figure 2B). Data on PhD completion during residency is not available; based on our results, we estimate this proportion to be around 10%-15% (Figure 2B).

Regarding scientific society membership, 5 of 18 initially enrolled residents (27.8%) withdrew, while 22 of 36 initially non-member residents (61.1%) joined by their last response (*P* < 0.01).

## Discussion

This longitudinal survey among French oncology residents highlights a significant decline in research interest and academic career aspirations over the past 4 years. Longitudinal follow-up reveals that even initially motivated residents often lose interest, reflecting growing disillusionment. Historical data further support this shift: a 2007 survey showed that 88% of French residents believed in viable academic career paths and 81% were interested in research.^7^

To place our findings in a broader perspective, we conducted a review of the international literature published over the last 10 years on research engagement and academic career trajectories across the oncology and physician-scientist training continuum (Supplementary Table S5, available at https://doi.org/10.1016/j.esmoop.2025.106025). Although our findings may initially appear context-specific, this comprehensive body of evidence strongly indicates that the observed trend reflects a global structural crisis affecting the oncology and physician-scientist workforce across all stages of training. The convergence of data from North America, Europe, the Middle East, and low- and middle-income countries demonstrates that the erosion of academic motivation is not driven by individual attitudinal change alone, but by systemic workforce constraints affecting education, funding, mentorship, protected research time, and career sustainability.

At the undergraduate level, studies consistently show that although interest in research remains high, actual participation is low and declines during training.^8^, ^9^, ^10^, ^11^, ^12^ Students across countries cite lack of time, insufficient methodological training, limited mentorship, and poor institutional visibility of research opportunities as the dominant barriers. Importantly, multiple cohorts show that as students progress through medical school, confidence in research skills decreases, perceived faculty encouragement diminishes, and enthusiasm for pursuing doctoral training drops. Early motivation alone is therefore insufficient to sustain research engagement unless it is reinforced by authentic, structured research experiences integrated into the core curriculum.

At the residency and fellowship level, the evidence is even more consistent.^13^^,^^14^ Across Europe, North America, and emerging academic systems, oncology trainees face a near-universal shortage of protected research time, combined with intensifying clinical workload, increasing administrative burden, and inadequate grant-writing support. Even in settings where residents actively participate in clinical trials and publications, satisfaction with research training remains modest. Exposure to clinical trial conduct during fellowship strongly predicts long-term research participation, yet such exposure remains unevenly distributed across institutions and practice settings. These structural barriers directly mirror the progressive disengagement observed in our French cohort. A major strength of our study is that it includes both medical oncology and radiation oncology residents, allowing a comprehensive representation of the oncology training continuum.

At the early career physician-scientist level, multiple national studies demonstrate a critical risk of attrition during the transition to junior faculty.^15^^,^^16^ Nearly half of early career physician-scientists in the United States consider leaving academia within 2 years, primarily due to burnout, work-family conflict, insufficient funding, lack of protected time, and under-compensation.^15^ Even among graduates of combined medical and doctoral programs, long-term research involvement declines outside a narrow set of specialties with protected academic infrastructures.^17^^,^^18^ Financial debt further compounds this attrition risk, especially for women. Collectively, these data demonstrate that the traditional physician-scientist pipeline is not failing because of lack of talent or motivation, but because of insufficient institutional protection during the most vulnerable career transitions.

At the system level, national workforce analyses show a dramatic contraction of the physician-scientist population, now representing only ∼1.5% of practicing physicians in some countries.^19^ The workforce is aging, time to independence continues to increase, and funding instability has become the norm rather than the exception.^20^ The persistent underrepresentation of women and socially disadvantaged groups across the research pipeline further weakens long-term workforce resilience.

Taken together, this global body of evidence indicates that the decline in research engagement observed in our cohort is not an isolated educational phenomenon, but rather the early manifestation of a downstream workforce bottleneck that ultimately threatens oncology innovation, clinical trial leadership, and translational progress. From a global policy perspective, these findings carry critical implications. Firstly, protected research time must be defined as a workforce policy standard rather than a discretionary institutional privilege. Secondly, research training must move from voluntary extracurricular exposure to formally integrated longitudinal pathways spanning undergraduate education, residency, and early faculty stages. Thirdly, mentorship must be systematically structured, evaluated, and incentivized. Fourthly, financial insecurity—including educational debt and unstable early career funding—must be directly addressed through coordinated governmental and institutional mechanisms. Finally, workforce planning must explicitly incorporate hybrid clinician-scientist career models that allow sustainable coexistence of clinical care, research, and personal life.

In this context, our French data reinforce international calls for coordinated, system-level action rather than isolated educational reforms. Without structural realignment of training, workload, funding, and career incentives, the global oncology workforce will continue to experience progressive academic attrition, with direct consequences for clinical trial capacity, innovation ecosystems, and patient access to research. The international evidence now clearly defines both the mechanisms driving attrition and the strategic policy levers required to reverse it. Addressing this challenge is no longer only an educational priority—it is a fundamental requirement for sustaining the future of oncology research and care worldwide.
