# Supplementary material for: Declining research interest among oncology residents: insights into the academic career from a nationwide longitudinal study in France
Source: ESMO Open. 2026 Jan 2;11(1):106025. doi: 10.1016/j.esmoop.2025.106025 (PMC12805360; doi:10.1016/j.esmoop.2025.106025)
Supplement: Supplementary Data [file mmc1.docx]

**Supplementary Table 1: Survey questions**

Questions marked with an asterisk (*) are multiple-choice questions.

| **Section 1: Demographics** |
| --- |
| 1. What is your gender? |
| 1. What is your age? |
| 1. What is your marital status? |
| 1. What is your residency city? |
| 1. What is your rank at the national matching exam? |
| 1. What is your predefined specialty choice? |
| 1. What was your desired specialty after the national matching exam? |
| **Section 2: Academic dimension** |
| 8. Do you know what translational research is? 9. What type(s) of research do you want to pursue during your career? * 10. Who motivated you to engage in fundamental research? * 11. Do you wish to pursue a master of science? 12. Do you wish to pursue a PhD? 13. Do you wish to pursue a fellowship? 14. What are your motivations for research? * 15. What are your barriers to conducting research? * 16. Do you want to publish in an international journal? 17. Do you want to present at a national or international congress? 18. Do you know what an impact factor is? |
| **Section 3: Clinical dimension** |
| 19. What is your average weekly hospital working time this year? 20. What is the average number of night shifts per month during the current academic year? 21. What is the average number of on-call duties weekends per semester? 22. Do you wish to pursue a complementary certification? 23. After completing your training, in which locations do you wish to specialize? * 24. What are your desired internship areas for free semesters? * 25. What motivates you to pursue oncology? * 26. What are your concerns regarding oncology? * 27. Have you ever considered opting out of the speciality in oncology? If yes, for what reasons? * |
| **Section 4: Training** |
| 28. How many hours of allocated to research time do you have per week? 29. How many hours of allocated to research time would you like to free up per week? 30. Do you have access to a half-day of supervised research per week? 31. Do you have access to a half-day of clinical or research training? 32. Do you find that the speciality courses organized by the faculty meet your needs? 33. What training materials would you like to have at your disposal? (Ranking of learning resources: 1 = most useful, 5 = least useful) 34. Would you like to have a mentor or guide? 35. If yes, who would you like as a mentor/guide? * 36. If yes, what aspects would you like your guidance to focus on? * |
| **Section 5: Life and career goals** |
| 37. What do you consider the ranking among money, social/personal life, research, and clinical work? (Ranking of life goals: 1 = most important, 4 = least important) 38. Do you want children? 39. Do you feel that your professional life prevents you from being personally fulfilled? If yes, to what extent? 40. What position are you looking for after your training? (Ranking of professional positions: 1 = most wanted, 6 = least wanted) |
| **Section 6: Associative involvement** |
| 41. Do you want to be involved in oncology associations? 42. Are you a member of one or more scientific societies or associations? * |

**Supplementary Table 2: Demographics**

|  | | **2020 (n=72)** | **2021 (n=95)** | **2022 (n=101)** | **2023 (n=109)** | ***p*** |
| --- | --- | --- | --- | --- | --- | --- |
| **Gender** | **Man** | 29 (40.3%) | 36 (37.9%) | 44 (43.6%) | 50 (45.9%) | 0.66 |
|  | **Woman** | 44 (61.1%) | 59 (62.1%) | 57 (56.4%) | 59 (54.1%) |  |
| **Age (median, IQR)** | | 24 (24,25) | 24 (24,25) | 24 (24,25) | 24 (24,25) | 0.98 |
| **Marital status** | **Single** | 45 (62.5%) | 57 (60.0%) | 57 (56.4%) | 75 (68.8%) | 0.47 |
|  | **Civil partnership** | 1 (1.4%) | 2 (2.1%) | 3 (3.0%) | 5 (4.6%) |  |
|  | **Married** | 1 (1.4%) | 0 (0.0%) | 2 (2.0%) | 1 (0.9%) |  |
|  | **Living with a partner** | 25 (34.7%) | 36 (37.9%) | 39 (38.6%) | 28 (25.7%) |  |
|  | **Divorced** | 0 (0.0%) | 0 (0.0%) | 0 (0.0%) | 0 (0.0%) |  |
| **City** | **Paris** | 10 (13.9%) | 19 (20.0%) | 19 (18.8%) | 17 (15.6%) | 0.69 |
|  | **Other** | 62 (86.1%) | 76 (80.0%) | 82 (81.2%) | 92 (84.4%) |  |
| **Rank at national matching exam** | **0-1500** | 41 (56.9%) | 45 (47.4%) | 50 (49.5%) | 49 (44.9%) | 0.55 |
|  | **1500-2500** | 17 (23.6%) | 23 (24.2%) | 21 (20.8%) | 23 (21.1%) |  |
|  | **> 2500** | 14 (19.4%) | 27 (28.4%) | 30 (29.7%) | 37 (33.9%) |  |
| **Predefined specialty choice** | **Medical Oncology** | 6 (8.3%) | 7 (7.4%) | 6 (5.9%) | 6 (5.5%) | 0.39 |
|  | **Radiation oncology** | 1 (1.4%) | 8 (8.4%) | 3 (3.0%) | 7 (6.4%) |  |
|  | **Unknown** | 65 (90.3%) | 80 (84.2%) | 92 (91.1%) | 96 (88.1%) |  |
| **Desired specialty after the national matching exam** | **Oncology** | 69 (95.8%) | 85 (89.5%) | 96 (95.0%) | 95 (87.2%) | 0.10 |
|  | **Other** | 3 (4.2%) | 10 (10.5%) | 5 (5.0%) | 14 (12.8%) |  |

**Supplementary Table 3: Evaluation of the training dimension**

|  | | **2020 (n=62)** | **2021 (n=92)** | **2022 (n=100)** | **2023 (n=107)** | ***p*** |
| --- | --- | --- | --- | --- | --- | --- |
| **Hours per week allocated to research time (median, IQR)** | | 4 (2, 5) | 2.5 (2, 4) | 2 (1, 4) | 2 (1, 3) | 0.001 |
| **Hours per week residents would like to free up (median, IQR)** | | 8 (5.625, 10) | 6 (4, 10) | 6 (4, 10) | 5 (4, 8) | 0.01 |
| **Access to half-day supervised research per week** | **Partially** | 24 (38.7%) | 26 (28.3%) | 28 (28.0%) | 32 (29.9%) | 0.07 |
|  | **Yes** | 14 (22.6%) | 19 (20.7%) | 27 (27.0%) | 40 (37.4%) |  |
|  | **No** | 25 (40.2%) | 47 (51.1%) | 45 (45.0%) | 35 (32.7%) |  |
| **Access to a half-day of clinical or research training** | **Partially** | 18 (29.0%) | 18 (19.6%) | 24 (24.0%) | 24 (22.4%) | 0.25 |
|  | **Yes** | 13 (21.0%) | 10 (10.9%) | 18 (18.0%) | 23 (21.5%) |  |
|  | **No** | 31 (50.0%) | 64 (69.6%) | 59 (59.0%) | 60 (56.1%) |  |
| **Courses adapted to needs (median, IQR)** | **0 = Not at all 100 = Completely** | 50 (25, 60) | 55 (35, 70) | 60 (38,75, 70) | 60 (45, 80) | 0.11 |
| **Ranking of available learning resources (mean)** | 1. **In-person faculty courses** 2. **E-Learning faculty courses** 3. **Hospital courses** 4. **Reference books** 5. **Guideline articles** | 1. Hospital Courses (2.00) 2. In-person faculty courses (2.68) 3. Reference books (3.22) 4. E-Learning faculty courses (3.24) 5. Guideline articles (3.86) | 1. Hospital Courses (2.13) 2. In-person faculty Courses (2.57) 3. E-Learning faculty courses (3.02) 4. Reference books (3.25) 5. Guideline articles (4.03) | 1. Hospital Courses (2.18) 2. In-person faculty courses (2.58) 3. E-Learning faculty courses (2.92) 4. Reference books (3.05) 5. Guideline articles (4.27) | 1. Hospital Courses (2.13) 2. In-person faculty courses (2.25) 3. E-Learning faculty courses (2.94) 4. Reference books (3.18) 5. Guideline articles (4.50) |  |
| **Desire to have a mentor** | **Already has one** | 1 (1.6%) | 1 (1.1%) | 5 (5.0%) | 3 (2.8%) | 0.64 |
|  | **Yes** | 59 (95.2%) | 84 (91.3%) | 90 (90.0%) | 96 (89.7%) |  |
|  | **No** | 2 (3.2%) | 7 (7.6%) | 5 (5.0%) | 8 (7.5%) |  |
| **If yes, desire for the mentor to be*** | **MD, PhD** | 9 (14.5%) | 12 (13.5%) | 10 (10.0%) | 13 (12.3%) | 0.52 |
|  | **Physician** | 3 (4.8%) | 4 (4.5%) | 5 (5.0%) | 9 (8.5%) |  |
|  | **Clinical fellow** | 19 (45.2%) | 32 (36.0%) | 27 (27.0%) | 33 (31.1%) |  |
|  | **Other intern** | 2 (3.2%) | 3 (3.4%) | 1 (1.0%) | 6 (5.7%) |  |
|  | **No preference** | 27 (43.5%) | 34 (38.2%) | 54 (54.0%) | 37 (34.9%) |  |
|  | **No need for guidance** | 2 (3.2%) | 4 (4.5%) | 3 (3.0%) | 8 (7.5%) |  |
| **If yes, desire for guidance to focus on*** | **Career (internships, post-internship)** | 56 (93.3%) | 71 (83.5%) | 83 (85.6%) | 90 (87.4%) | 0.67 |
|  | **Human aspect** | 37 (61.7%) | 37 (43.5%) | 60 (61.9%) | 60 (58.3%) |  |
|  | **Teaching aspect** | 46 (76.7%) | 66 (77.6%) | 66 (68.0%) | 79 (76.7%) |  |
|  | **Research aspect** | 35 (58.3%) | 54 (63.5%) | 52 (53.6%) | 47 (45.6%) |  |
| **Desire to engage in associative activities** | **Yes** | 14 (22.6%) | 17 (18.5%) | 14 (14.0%) | 16 (15.0%) | 0.68 |
|  | **No** | 10 (16.1%) | 15 (16.3%) | 21 (21.0%) | 25 (23.4%) |  |
|  | **Maybe later** | 38 (61.3%) | 60 (65.2%) | 65 (65.0%) | 66 (61.7%) |  |
| **Member of a scientific society or association*** | **No** | 35 (56.5%) | 62 (67.4%) | 69 (69.0%) | 80 (74.8%) | 0.01 |
|  | **AERIO** | 25 (40.3%) | 24 (26.1%) | 22 (22.0%) | 24 (22.4%) |  |
|  | **SFjRO** | 7 (11.3%) | 2 (2.2%) | 7 (7.0%) | 5 (4.7%) |  |
|  | **ESTRO** | 0 (0.0%) | 1 (1.1%) | 0 (0.0%) | 1 (0.9%) |  |
|  | **ESMO** | 1 (1.6%) | 6 (6.5%) | 2 (2.0%) | 4 (3.7%) |  |
|  | **Other** | 0 (0.0%) | 8 (8.7%) | 9 (9.0%) | 2 (1.9%) |  |

**Supplementary Table 4: Life and career goals**

|  | | **2020 (n=62)** | **2021 (n=92)** | **2022 (n=100)** | **2023 (n=107)** | ***p*** |
| --- | --- | --- | --- | --- | --- | --- |
| **Ranking by order of priority (average)** | 1. **Money** 2. **Social and personal life** 3. **Research** 4. **Clinical practice** | 1. Social and personal life (1.37) 2. Clinical practice (2.11) 3. Money (2.94) 4. Research (3.59) | 1. Social and personal life (1.37) 2. Clinical practice (2.02) 3. Money (3.05) 4. Research (3.55) | 1. Social and personal life (1.43) 2. Clinical practice (1.99) 3. Money (2.89) 4. Research (3.69) | 1. Social and personal life (1.40) 2. Clinical practice (2.00) 3. Money (2.84) 4. Research (3.75) | 0.81 |
| **Do you want children?** | **Yes** | 49 (79.0%) | 81 (88.0%) | 72 (72.0%) | 68 (63.6%) | 0.005 |
|  | **No** | 3 (4.8%) | 2 (2.2%) | 3 (3.0%) | 3 (2.8%) |  |
|  | **Maybe** | 8 (12.9%) | 8 (8.7%) | 24 (24.0%) | 26 (24.3%) |  |
|  | **Yes, but it seems incompatible with professional activity** | 2 (3.2%) | 1 (1.1%) | 1 (1.0%) | 9 (8.4%) |  |
|  | **Already had children** | 0 (0.0%) | 0 (0.0%) | 0 (0.0%) | 1 (0.9%) |  |
| **Does professional life interfere with personal fulfillment; if yes, to what extent (median, IQR)** | **Yes** | 15 (24.2%) | 17 (18.5%) | 28 (28.0%) | 22 (20.6%) | 0.41 |
|  | **No** | 47 (75.8%) | 75 (81.5%) | 72 (72.0%) | 85 (79.4%) |  |
|  | **0 = No at all**  **100 = Completely** | 25 (5, 45) | 25 (10, 40) | 30 (15, 55) | 30 (15, 65) | 0.03 |
| **Ranking of desired positions after training** | 1. **University public hospital** 2. **Non-university public hospital** 3. **Private center** 4. **Cancer care center** 5. **Industry** 6. **Research units** | 1. Cancer care center (1.63) 2. Private center (2.67) 3. University public sector (2.78) 4. Non-university public sector (3.41) 5. Industry (5.14) 6. Research units (5.74) | 1. Cancer care center (1.80) 2. Private center (2.58) 3. Non-university public sector (2.93) 4. University public sector (3.03) 5. Industry (5.16) 6. Research units (5.49) | 1. Cancer care center (2.00) 2. Private center (2.57) 3. University public sector (2.78) 4. Non-university public sector (3.04) 5. Industry (5.08) 6. Research units (5.53) | 1. Cancer care center (1.96) 2. Private center (2.51) 3. University public sector (2.74) 4. Non-university public sector (3.13) 5. Industry (5.18) 6. Research units (5.48) | 0.74 |

**Supplementary Table 5: International studies on research engagement across the oncology and physician–scientist training continuum.**

| **Reference** | **Year** | **Location** | **Target population** | **Key findings** | **Barriers identified** | **Key recommandations** |
| --- | --- | --- | --- | --- | --- | --- |
| Amgad et al.(1) | 2015 | International | Undergraduate medical students | About **72%** of medical students reported interest in performing research, yet only **31%** expressed interest in a research‑career, and just **12%** intended “significant” research commitment.  Participation in research during medical school was associated with more than double the odds of subsequent academic career success (OR > 2). | Financial barriers: cost of intercalated degrees, fear of lost income, and low perceived salaries of academic careers.  Lack of mentorship and limited availability of engaged supervisors.  Time constraints due to heavy academic workload and limited flexibility in curricula.  Administrative and ethical approval burdens.  Weak research infrastructure, especially in developing countries: insufficient information technology systems, poor laboratory resources, high student-to-mentor ratios.  Gender gap in publication output (men more likely to publish), not explained by differences in attitudes or interest.  Students often perceive research as primarily a requirement for securing competitive residency positions rather than intellectual curiosity. | Integrate structured and flexible research pathways within curricula.  Expand mentorship programs.  Provide financial support, stipends, or reduced tuition for research degrees or extended research time.  Reduce bureaucratic barriers by simplifying institutional review and ethical approval processes for student projects.  Strengthen research infrastructure, especially in resource-limited settings, with improved databases, laboratory access, and research governance.  Promote early exposure to research and cultivate curiosity through authentic research tasks and skills training.  Encourage medical schools and national bodies to align research involvement with pathways for career advancement, not only residency competitiveness. |
| Pathipati et al.(2) | 2016 | USA | Medical students (MD only), all years | **61% of students had taken or planned to take a research year; significantly more common among students entering highly competitive specialties.**  **The most frequent primary reason for taking a research year was to increase competitiveness for residency (32%).**  **Other common motivations: time to pursue other opportunities (24%) and academic interest (23%).**  **35% of students would not take a research year if guaranteed a residency position of their choice (only 41% in competitive specialties would still take one).**  **Students often used the “research year” to pursue unrelated interests (writing, policy, personal projects) rather than genuine scientific research.** | Students feel pressured by residency selection processes.  Low educational value: many projects are felt poorly supervised, or designed mainly to generate publications.  Workload and stress during clinical years lead some students to use the research year as recovery time.  Institutional culture encourages research even when students lack interest or adequate preparation. | Realign incentives so that students engage in research for education and curiosity, not only residency competitiveness.  Provide meaningful alternatives to research years for personal development (policy, global health, entrepreneurship).  Ensure better supervision and support to improve the educational value and quality of student research.  Reassess how residency programs evaluate applicants to reduce pressure for high publication counts.  Conduct national-level evaluations to clarify the role and purpose of research years in medical education. |
| Kharraz et al.(3) | 2016 | Saudi Arabia | Undergraduate medical students | Participation in research differed significantly by gender (males 68.6% vs females 45.4%). | Lack of time due to heavy academic workload.  Lack of structured research courses and insufficient curriculum integration.  Limited availability of research mentors and difficulty finding appropriate supervisors.  Scarcity of research opportunities and projects accessible to students.  Gender-related barriers, especially for female students seeking same-gender mentorship. | Integrate structured and mandatory research courses into the curriculum.  Increase availability of mentors and create mentorship programs.  Expand institutional research opportunities and ensure clear pathways for participation.  Allocate protected time for research or reduce workload during research periods.  Encourage faculty engagement and recognition of student research supervision. |
| Moraes et al.(4) | 2016 | Brazil | Undergraduate medical students  From first to sixth year | 81.7% of students reported interest in research during medical school, and 60.8% planned to continue research after graduation.  Only 4.7% considered research the most important component of medical training; 77.7% ranked clinical practice first.  Interest in research was uniform across all six years, with no significant differences by age, gender, family background, or previous university degrees.  Around 10% already had at least one publication.  Students expressed strong conceptual appreciation for research but weak integration into actual training, reflecting a mismatch between motivation and curricular structure. | Research is treated as optional and voluntary rather than integrated into the curriculum.  Heavy emphasis on clinical practice and theory leaves little time for research activities.  Students lack early guidance, structured supervision, and continuous support throughout the degree.  Sociocultural perception of physicians as clinicians rather than investigators reduces aspiration toward research careers.  Introductory research methodology courses alone are insufficient to stimulate lasting research engagement. | Introduce longitudinal research training, beginning early in the curriculum.  Provide structured supervision, continuous mentoring, and clearly defined research goals across training stages.  Strengthen research culture within medical education by promoting the value of investigative practice alongside clinical skills.  Develop institutional incentives and national programs to support student research engagement and academic career development.  Encourage partnerships across health sciences disciplines to expose students to active research environments. |
| Sidiqi et al.(5) | 2019 | USA | Medical students applying to radiation oncology | About one-third of applicants took a gap year during medical school, mainly to increase research productivity.  The main motivation for taking a gap year was to produce more publications, while a smaller proportion used the year to obtain an additional academic degree.  Half of all applicants believed that taking a gap year was important or very important for successfully matching into radiation oncology.  Most students who took a gap year found it to be productive and beneficial for improving their chances of matching.  The strongest predictor of taking a gap year was having fewer publications before the gap year.  Taking a gap year was not associated with higher examination scores and was not clearly linked to better match success. | Major financial burden: most students relied on personal savings, family support, or loans to fund their gap year.  Difficulty obtaining external funding was reported by the large majority of applicants.  Some students avoided a gap year because they were unable or unwilling to delay the start of residency training.  Others reported they already had sufficient research experience and therefore did not feel a gap year was necessary.  The pressure to take a gap year may discourage students without financial resources from applying to radiation oncology. | Increase accessible external funding opportunities to reduce financial inequality linked to gap years.  Create centralized, publicly available listings of gap year research opportunities.  Reassess how residency programs value research output to ensure that gap years are not an implicit requirement.  Encourage meaningful research engagement within the standard medical school curriculum, rather than relying on gap years.  Monitor possible long-term effects on workforce diversity and access to competitive specialties. |
| Ommering  et al.(6) | 2019 | Netherlands | Undergraduate medical students  First-year medical students followed prospectively in their second year | Students with higher intrinsic motivation at entry were far more likely to engage in research during the second year.  Extrinsic motivation showed only a weak association and lost significance after adjusting for other factors.  Only 17.5% of students engaged in research activities during year 2, despite high initial motivation scores. | Limited time and limited opportunities for research during early training.  Research involvement dependent on voluntary extracurricular programs rather than integrated pathways.  Heavy academic workload limits capacity to participate in research.  Students often see research as time-consuming and difficult to combine with the curriculum. | Strengthen intrinsic motivation early by providing autonomy, meaningful research tasks, and strong role models.  Integrate authentic research experiences into the curriculum rather than relying on voluntary programs.  Support self-efficacy through structured early research training and supervised projects.  Create supportive research environments where students can participate in real clinical or laboratory work. |
| Ommering  **et al.(7)** | 2021 | Netherlands | Undergraduate medical students  First-year medical students followed prospectively in their second year | Success in authentic research tasks was linked to higher intrinsic motivation for research one year later.  After adjusting for baseline motivation and early academic performance, only success in orally presenting research remained a significant predictor of increased intrinsic motivation.  Presenting research also led to higher research self-efficacy, even after adjusting for baseline self-efficacy.  Success on the written exam had no effect on intrinsic motivation, extrinsic motivation, or self-efficacy.  None of the success experiences influenced extrinsic motivation.  Findings support social cognitive theory: mastery and positive performance experiences strengthen confidence and interest in research. | Standard exams do not reinforce research identity or perceived competence because they are not part of the real research process.  Written research reports generated delayed, non-interactive feedback, limiting impact on self-efficacy.  Students often consider writing challenging, reducing the sense of mastery despite good performance.  Early students may not yet associate research skills with career advancement, reducing extrinsic motivation. | Integrate authentic research assessments (oral presentation, research report, real data analysis) into early medical curricula.  Prioritize oral research presentations,  Provide real-time feedback dialogue to enhance learning and increase perceived competence.  Create structured opportunities for students to engage in real research tasks to foster motivation and early research involvement.  Consider educational design principles from social cognitive theory to strengthen self-efficacy, especially for students from backgrounds underrepresented in academia. |
| Roche et al.(8) | 2021 | United Kingdom | Undergraduate medical students | Research involvement during medical school is linked to later academic success, but opportunities vary widely across institutions.  45% of respondents reported involvement in some form of research. Students who expressed a future interest in research careers had significantly higher odds of current research involvement (adjusted OR ≈ 2.3). | Variation between medical schools in access to research projects, curriculum requirements, and support for intercalated degrees.  Limited previous exposure to research.  Inconsistent availability of mentors.  Perception that research is no longer rewarded in postgraduate selection systems.  Time constraints and competing clinical demands during training. | Medical schools should monitor student attitudes toward research and adjust curricula to strengthen early exposure.  Improve access to mentors, research networks, and structured research pathways.  Enhance communication about the value of research experience for clinical practice and long-term career development.  Encourage national bodies to consider how policy changes (such as scoring systems) influence student engagement with research.  Use findings to inform national strategies aimed at strengthening clinical academic pathways. |
| Sobczuk et al.(9) | 2022 | Poland | Undergraduate medical students (2^nd^ and 5^th^‑year) | Over half of students reported strong scientific interest during high school, but only one-third plan to pursue research after graduation.  Participation in student scientific groups was high, yet actual involvement in research projects remained low (8.5% in second year, 35% in fifth year).  Students with strong prior scientific interest were more likely to join research groups, participate in projects, and consider research careers.  Fifth-year students perceived university attitudes toward research as less supportive and reported lower enthusiasm for acquiring research skills.  Research methodology training was considered insufficient, and promotion of research opportunities inadequate. | Lack of time, lack of knowledge on how to begin research, and lack of funding or resources.  Limited experience and insufficient training in research methods.  Poor visibility of research opportunities and perceived discouragement from some faculty.  Difficulty balancing research with academic workload.  Very low participation in international conferences or external research programs. | Integrate structured research methodology earlier in the curriculum and expand dedicated coursework.  Improve promotion of research opportunities and ensure better institutional support.  Offer tailored research pathways for students with strong scientific motivation from high school.  Provide mentorship programs, financial support, and access to international exchanges.  Develop flexible curricula that accommodate both clinically oriented students and research-oriented students. |
| Sanabria‑de la Torre et al.(10) | 2023 | Spain | Undergraduate medical students  2nd, 4th, and 6th year | Fourth‑ and sixth‑year students were more likely to show **no interest in research** (50.4% vs 28.1% for second‑years,) and less likely to want to pursue a doctoral thesis (75.0% vs 50.9%).  Skills confidence decreases with progression: more 4th/6th-year students felt they lacked the skills to do research (52% vs. 19% in 2nd year).  Many students perceive that professors do not encourage research (74.6% of 4th/6th-year students vs. 40.6% in 2nd year).  Participation in scientific events was very low.  Students recognize that research is essential for medical progress but feel it is poorly integrated into training. | Lack of funding (reported by 97.8% of students).  Lack of awareness of research opportunities (80%).  Feeling insufficiently skilled, especially among senior students.  Perception that research may negatively impact academic performance, delay clinical practice, or reduce leisure time.  Limited encouragement from faculty and insufficient integration of research into courses.  Very low participation in dissemination events and limited early exposure to hands-on research. | Provide early research exposure, mentorship programs, and structured integration of research into the curriculum.  Increase visibility of research opportunities and improve communication between faculty and students.  Develop initiatives to support student participation in scientific events and promote a research-friendly culture.  Expand research methodology courses and provide training to improve skills and confidence.  Offer incentives, scholarships, and exchange programs to promote high-quality student research. |
| Abusamak et al.(11) | 2024 | Jordan | Undergraduate medical students  From second to sixth year | Students showed positive attitudes toward research: over 80% agreed research helps learning and should be part of the curriculum.  Knowledge levels were low, but increased steadily with advancement in academic year and with age; senior students performed best.  Women had higher knowledge scores and slightly more positive attitudes than men.  Students aiming for residency abroad showed more positive attitudes toward research, likely reflecting international application expectations.  Research participation increased with academic seniority but dropped temporarily during the demanding clinical year.  Only half agreed that a research track record should influence residency selection. | Insufficient training in research methods (most cited barrier, >80%).  Lack of research opportunities and lack of mentorship/support from faculty (over 70%).  Inadequate infrastructure and funding, including limited research facilities and financial support.  Insufficient time due to heavy course load and clinical responsibilities.  Some students reported limited availability of faculty members to supervise research. | Integrate structured research training early into the medical curriculum.  Provide mentorship programs, improved supervision, and faculty development in research guidance.  Expand research opportunities, facilities, and protected time for student research.  Offer financial support for student-led projects to remove economic barriers.  Encourage a research culture by recognizing and rewarding student research achievements, especially for those considering international career pathways. |
| Penel et al.(12) | 2025 | France | Undergraduate medical students | Only 7.3 percent of students considered oncology as a first, second, or third specialty choice, showing that oncology remains a low-attractiveness specialty.  Hospital rotation in oncology was the strongest predictor of choosing oncology as a specialty:  Students who completed a rotation in a medical oncology unit were nine times more likely to choose oncology.  Students who completed a rotation in a radiation oncology unit were five times more likely to choose oncology.  Identification with a physician practicing oncology also significantly increased the likelihood of choosing the specialty.  Motivations differed by gender:  Among men, interest in fundamental research was key positive factor.  Among women, interest in long-term relationships with patients, and hospital-based careers were the main positive factors.  Students interested in immediate treatment results or emergency care were less likely to choose oncology. | Limited exposure to oncology during medical training, reducing student awareness and interest.  Low visibility and attractiveness of oncology compared with other specialties.  Perceived lack of immediate treatment results, particularly for students attracted to acute care.  Work–life balance concerns, especially among female students.  Lower attractiveness of oncology for students wishing to practice in rural areas.  Possible selection bias and regional limitation, affecting generalizability. | Increase and strengthen mandatory clinical rotations in both medical oncology and radiation oncology during medical school.  Promote role modelling and mentorship by practicing oncologists to enhance identification with the specialty.  Develop targeted communication and orientation campaigns to improve the image and attractiveness of oncology.  Tailor recruitment strategies according to gender-specific motivations.  Integrate early exposure to oncology research and long-term patient care into undergraduate training.  Anticipate future oncology workforce needs through early, structured specialty orientation policies. |
| Mahmood et al.(13) | 2025 | Pakistan | Undergraduate final-year medical and dental students | Very few students were actively involved in research, even though most agreed that research is important for learning and evidence-based practice.  Students reported that the research component in the curriculum is superficial and too limited to build real skills.  Many students felt research is not valued by the institution, is not rewarded, and competes directly with the main priority of passing exams and clinical assessments. | Heavy clinical workload leaving almost no time for research.  Lack of external incentives (no recognition, rewards, or institutional encouragement), and low awareness of how research could benefit careers.  Insufficient training in research methods, statistics, and practical research skills, making projects feel too difficult to start.  Inadequate financial support, outdated or poorly maintained laboratories, and limited access to journals and databases.  Limited mentorship: teachers are overloaded with teaching and private practice and have little time or institutional support to supervise student research. | Integrate structured, practical research training and mandatory research components into the undergraduate curriculum, with explicit time allocated for research.  Provide better institutional recognition, rewards, and local opportunities to present or publish student work to increase motivation.  Improve financial and infrastructural support, including access to laboratories, online databases, and help with publication costs.  Establish formal mentorship programmes, assigning research mentors and supporting faculty with time and incentives for supervision.  Implement broader institutional and policy reforms to create a culture where research is seen as a core part of medical education, not an optional extra. |
| Skinnider MA et al.(14) | 2018 | Canada | Graduates of MD/PhD programmes | The majority of graduates remained significantly involved in research based on at least one indicator of research activity.  Factors positively associated with continued research involvement were:  Completion of a dedicated research fellowship after combined training.  A higher number of first-author or co-author publications during training.  Longer duration of combined medical and doctoral training.  Factors negatively associated with sustained research involvement were:  Completion of a master’s degree before entering combined training.  Female gender.  Educational debt.  Pursuit of a clinical specialty outside internal medicine, paediatrics, neurology, pathology, and surgical specialties.  Although most graduates remained active in research, many did not occupy classical physician–scientist roles | High educational debt, which negatively impacts long-term research engagement.  Lack of additional structured research training during residency, limiting transition to sustained research careers.  Gender-related factors contributing to lower long-term research participation among women.  Specialty-specific constraints, with certain clinical fields offering less opportunity for protected research time.  Limited stability of long-term research funding following completion of training. | Combined training programs should prioritize research productivity during training, particularly publication output.  Trainees should be actively encouraged to pursue additional research fellowships during residency.  Policymakers should implement stable funding mechanisms to reduce educational debt for physician–scientists.  Targeted strategies are needed to reduce attrition among women in physician–scientist career pathways.  Long-term workforce planning should support sustained research engagement beyond completion of formal training. |
| Andriole et al.(15) | 2021 | USA | Graduates of MD–PhD programs | The majority of graduates (78%) reported being engaged in research after completing training.  On average, those engaged in research devoted about half of their professional time to research activities.  Engagement in research varied strongly by medical specialty: the highest research involvement was observed in internal medicine, pediatrics, neurology, psychiatry, and pathology, while lower involvement was seen in surgical and imaging-related specialties.  Graduates who completed at least one dedicated research year during residency were much more likely to remain engaged in research later in their careers and devoted more time to research.  Graduates who intended to pursue full-time academic research careers at the time of graduation showed substantially higher long-term research engagement.  Educational debt at graduation was not a major predictor of later research engagement. | Lack of protected research time in several clinical specialties reduces long-term research engagement.  Residency structures in procedure-heavy specialties leave little room for sustained research activity.  Women showed slightly lower long-term research engagement than men.  Training environments with fewer institutional research resources produced lower research engagement.  Transitions between medical school, residency, and early faculty positions represent vulnerable periods for attrition from research careers. | Expand and protect dedicated research time during residency to sustain long-term research careers.  Strengthen institutional cultures that support combined clinical and research careers across a broader range of specialties.  Improve continuity of mentorship from medical school through residency and early faculty positions.  Provide targeted support for early-career physician–scientists during critical transition periods.  Use these national outcome data to guide workforce policy and specialty-specific research pathways. |
| Dahn et al.(16) | 2020 | Canada | Radiation oncology residents | 66% of residents felt there was a lack of protected research time, compared to only 20% of program directors.  While 94% of residents valued mentorship, only 48% actually had a mentor.  High stress linked to research expectations and felt research involvement was crucial for obtaining competitive jobs or fellowships. | Lack of protected research time for residents and supervising faculty.  High clinical workload.  Insufficient mentorship.  Limited training in methodology, statistics, and scientific writing.  Difficulty accessing high-quality projects and navigating ethics approval.  Too many competing educational activities within the curriculum.  Limited institutional funding and inadequate statistical support. | Increase protected time for both residents and faculty mentors dedicated to research and scholarly activity.  Strengthen mentorship programs and ensure mentors are accessible, trained, and supportive.  Implement structured, longitudinal research curricula that include training.  Improve departmental support by providing lists of available projects and supervisors.  Reduce competing demands in the curriculum and rebalance workload to facilitate resident engagement in meaningful research.  Foster alignment between residents and program directors regarding expectations for academic productivity and career planning. |
| Wang et al.(17) | 2021 | Canada | Radiation oncology residents | Most residents were highly satisfied with both their specialty choice and their training program.  The majority planned to pursue post-residency fellowships, mainly to improve competitiveness in the job market, gain specific clinical expertise, and pursue research interests.  Only a minority felt confident they could obtain a staff position in their preferred disease site or geographic location.  Residents perceived the current job market as less competitive than in the past and expected it to improve in the future.  Higher educational debt was associated with greater stress, poorer work–life balance, and higher psychological burden.  Most residents favored employment in academic hospital centers. | Persistent anxiety related to employment opportunities after graduation.  Heavy clinical workload and examination pressure.  High educational debt, worsening stress and work–family balance.  Limited confidence in securing preferred jobs or locations.  Insufficient career mentoring focused on job market competitiveness.  Discrepancy between training satisfaction and uncertainties about long-term employment. | Strengthen career mentoring and employment guidance during residency.  Provide targeted support for residents with high educational debt to reduce wellness risks.  Improve job market transparency and national workforce planning.  Enhance structured support for post-residency fellowship pathways and research-oriented careers.  Integrate wellness-focused strategies within competency-based medical education reforms. |
| Karamayan et al. (18) | 2025 | Armenia | Medical oncology residents | Most residents reported low to moderate confidence in interpreting scientific literature, indicating limited research literacy.  Although the majority expressed a strong desire to participate in research, only a small proportion felt they knew how to initiate a research project.  A large majority of residents spent most of their time on clinical duties, with very limited time dedicated to education or research activities.  Interest in journal clubs was very high, with most residents expressing strong enthusiasm for regular sessions.  Residents were particularly interested in learning how to interpret scientific articles, write case reports, and stay updated with practice-changing research.  Most residents stated they would complete short educational assignments if the workload remained manageable. | Lack of time due to heavy clinical workload.  High clinical service demands, leaving little room for educational or research activities.  Limited mentorship and a non-motivating academic environment.  Insufficient familiarity with research methodology and project initiation.  Lack of structured opportunities for scholarly engagement within the residency curriculum. | Formalise journal clubs within the oncology residency curriculum as a core educational activity.  Provide structured training in research methods, scientific writing, and literature interpretation.  Create protected time for educational and scholarly activities alongside clinical duties.  Strengthen mentorship structures  Use journal clubs as a platform to build a sustainable culture of inquiry and academic development among oncology residents in Armenia. |
| Kirkwood et al. (19) | 2024 | USA | Medical oncology fellows | Nearly 60% of fellows planned to participate frequently in clinical trials after completing training (twice as likely if fellows had frequent exposure to clinical trial enrollment and management during fellowship compared with those with limited exposure).  Fellows who expected to work in academic practice were far more likely to plan active research involvement than those planning non-academic careers.  Nearly half of fellows planned to take on research leadership roles, such as writing trial protocols, applying for research funding, or serving as principal investigators.  Fellows with frequent exposure to research leadership activities during fellowship were much more likely to anticipate future leadership roles.  Anticipated research participation did not differ by training year, suggesting that exposure, rather than seniority, drives future engagement. | Insufficient exposure to research and clinical trial conduct during fellowship training.  Feeling underprepared to conduct research independently after training.  Lower research expectations and opportunities in non-academic practice settings.  Limited opportunities for structured leadership experience in clinical research for some trainees. | Increase structured exposure to clinical trial enrollment, management, and leadership activities during fellowship training.  Ensure that all fellows, regardless of intended career path, receive hands-on experience in clinical research.  Strengthen partnerships between academic and community training environments to expand research exposure.  Integrate clinical trial leadership training into fellowship curricula.  Use enhanced research exposure during training as a strategy to improve patient access to clinical trials in routine practice. |
| Farhat et al. (20) | 2025 | USA | Early-career physician-scientists: residents, fellows, junior faculty | 49% considering leaving academic medicine within 2 years.  Major reasons: burnout (35%), stress (35%), funding challenges (30%), under-compensation (17%).  Top challenges: clinical–research balance (63%), work–family conflict (53%), insufficient funding (41%).  Only 43% received a career development award; 52% face restrictions on protected time.  Strong preference for hybrid research–clinical roles with work-life balance and financial security. | Burnout, stress, and work–life imbalance.  Insufficient protected research time; heavy clinical burdens.  Funding scarcity and low early-career award success rates.  Under-compensation, region-specific inequities.  Persistent racial and ethnic underrepresentation.  Limited institutional incentives (e.g. salary equity, childcare support). | Expand early-career funding and bridge support mechanisms.  Guarantee minimum protected research time through institutional policy.  Develop structured mentorship and tailored support networks for underrepresented minority trainees.  Improve salary support, childcare resources, and workload distribution.  Strengthen advocacy organizations to support retention.  Implement coordinated institutional and governmental strategies for workforce sustainability. |
| Garrison et al.(21) | 2014 | USA | Early-career physician–scientist | The number of early-career physician–scientists holding independent national research funding has stagnated despite growth in the overall biomedical workforce.  The average age at first major independent research grant increased steadily, delaying research independence.  Early-career physician–scientists face prolonged training periods, delayed financial stability, and increased dependence on soft money.  Funding success is increasingly concentrated among older investigators, limiting renewal of the workforce. | Declining success rates for first-time national research funding applications.  Delayed access to independent funding, prolonging career insecurity.  High educational debt at the completion of training.  Instability of early-career funding mechanisms.  Rising clinical workload that competes with research time.  Misalignment between training duration and funding timelines. | Protect and expand early-career targeted national research funding mechanisms.  Shorten the time to first independent research grant.  Strengthen bridge funding programs between training and first faculty appointment.  Reduce educational debt burden for physician–scientists through national policies.  Improve job security and institutional support during the transition to independence. |
| Cammarota et al.(22) | 2025 | Europe (majority from Italy, Netherlands, Spain, Turkey, Germany, UK). | Young and early-career oncologists  (within or ≤10 years after specialty training) in gastrointestinal oncology | Most respondents worked mainly in clinical roles, with over 75% reporting little to no dedicated research time.  75% faced significant challenges conducting and publishing research.  Main obstacles: lack of protected time (77%), limited funding (48%), and insufficient support for grant writing (47%).  Nearly half reported well-being and workload issues negatively affecting research productivity.  Strong demand for structured training, mentorship, research infrastructure, and opportunities to join clinical trials and collaborative projects. | Heavy clinical workload with minimal protected research time.  Limited funding and inadequate support for preparing or managing grant applications.  Lack of institutional well-being policies, excessive workload, and burnout.  Gender-related obstacles more frequently reported by women.  Unequal access to research opportunities across countries and institutions. | Develop structured training programs, mentorship frameworks, and research support services.  Improve access to funding and provide guidance for grant development and management.  Implement well-being, workload, and resilience measures within institutions.  Expand opportunities for involvement in clinical trials, collaborative networks, and interdisciplinary research.  Strengthen platforms to equalize access to research pathways across Europe. |
| Gerber et al.(23) | 2025 | USA | Academic oncology physicians, including clinical research physicians, laboratory-based physician researchers, and clinical educators | Start-up financial support and protected research time showed large institutional variability, with clinical research physicians generally receiving less financial support and shorter support duration than laboratory-based physician researchers.  Clinical workload also varied substantially between institutions, with wide differences in outpatient and inpatient responsibilities.  Over recent years, inpatient clinical service requirements decreased, while outpatient clinical work remained stable.  Institutions increasingly considered clinical productivity when evaluating the success of clinical research physicians.  The sources used to maintain protected research time became more diverse over time, with growing reliance on institutional and philanthropic support rather than external research funding alone.  The environment of clinical research was significantly affected by the period of the COVID-19 pandemic, with additional operational and financial pressure on both institutions and faculty.  Availability of dedicated research resources (core labs/institutional funding) was associated with higher research productivity. | Insufficient and short-term protected research time, which is often misaligned with the long timelines required for developing and conducting investigator-initiated clinical trials.  Lower start-up financial support for clinical research physicians compared with laboratory-based physician researchers.  Increasing clinical productivity expectations, which compete directly with research responsibilities.  High clinical workload, particularly in inpatient services at some centers.  Dependence on unstable funding sources to support protected research time and salaries.  Ongoing burnout and workforce attrition risks, amplified by pandemic-related stress and long-standing financial pressures. | Establish more stable and longer-term institutional support for protected research time for clinical research physicians.  Align start-up support with the real costs and timelines of investigator-initiated clinical trials.  Rebalance expectations between clinical productivity and research output to avoid undermining research careers.  Strengthen national and institutional policies that protect clinical research time as a core mission of cancer centers.  Increase coordination between departments, cancer centers, and health systems to ensure sustainable support models.  Prioritize long-term workforce retention strategies to prevent further decline in academic clinical research careers. |

^1. Amgad M, Man Kin Tsui M, Liptrott SJ, Shash E. Medical Student Research: An Integrated Mixed-Methods Systematic Review and Meta-Analysis. PLoS One. 2015;10(6):e0127470.^

^2. Pathipati AS, Taleghani N. Research in Medical School: A Survey Evaluating Why Medical Students Take Research Years. Cureus. 2016;8(8):e741.^

^3. Kharraz R, Hamadah R, AlFawaz D, Attasi J, Obeidat AS, Alkattan W, et al. Perceived barriers towards participation in undergraduate research activities among medical students at Alfaisal University-College of Medicine: A Saudi Arabian perspective. Med Teach. 2016;38 Suppl 1:S12-8.^

^4. Moraes DW, Jotz M, Menegazzo WR, Menegazzo MS, Veloso S, Machry MC, et al. Interest in research among medical students: Challenges for the undergraduate education. Rev Assoc Med Bras (1992). 2016;62(7):652-8.^

^5. Sidiqi B, Gillespie EF, Wang C, Dawson M, Wu AJ. Mind the Gap: An Analysis of "Gap Year" Prevalence, Productivity, and Perspectives Among Radiation Oncology Residency Applicants. Int J Radiat Oncol Biol Phys. 2019;104(2):456-62.^

^6. Ommering BWC, van Blankenstein FM, Wijnen-Meijer M, van Diepen M, Dekker FW. Fostering the physician-scientist workforce: a prospective cohort study to investigate the effect of undergraduate medical students' motivation for research on actual research involvement. BMJ Open. 2019;9(7):e028034.^

^7. Ommering BWC, van Blankenstein FM, van Diepen M, Dekker FW. Academic Success Experiences: Promoting Research Motivation andSelf-Efficacy Beliefs among Medical Students. Teach Learn Med. 2021;33(4):423-33.^

^8. Roche S, Bandyopadhyay S, Grassam-Rowe A, Brown RA, Iveson P, Mallett G, et al. Cross-sectional Survey of Medical student Attitudes to Research and Training pathways (SMART) in the UK: study protocol. BMJ Open. 2021;11(9):e050104.^

^9. Sobczuk P, Dziedziak J, Bierezowicz N, Kiziak M, Znajdek Z, Puchalska L, et al. Are medical students interested in research? - students' attitudes towards research. Ann Med. 2022;54(1):1538-47.^

^10. Sanabria-de la Torre R, Quiñones-Vico MI, Ubago-Rodríguez A, Buendía-Eisman A, Montero-Vílchez T, Arias-Santiago S. Medical students' interest in research: changing trends during university training. Front Med (Lausanne). 2023;10:1257574.^

^11. Abusamak M, AlQato S, Alrfooh HH, Altheeb R, Bazbaz L, Suleiman R, et al. Knowledge, attitudes, practices and barriers of medical research among undergraduate medical students in Jordan: a cross-sectional survey. BMC Med Educ. 2024;24(1):23.^

^12. Penel N, Cren PY, Ducroq C, Laurent EMN, Choukroun G, Hazzan M, et al. Motivational factors influencing the choice of oncology as a specialty among French medical students. BMC Med Educ. 2025;25(1):447.^

^13. Mahmood A, Rehman N, Huang X, Riaz I. Barriers to undergraduate medical students' research engagement in Pakistan: a qualitative exploration. BMC Med Educ. 2025;25(1):592.^

^14. Skinnider MA, Twa DDW, Squair JW, Rosenblum ND, Lukac CD. Predictors of sustained research involvement among MD/PhD programme graduates. Med Educ. 2018;52(5):536-45.^

^15.Andriole DA, Grbic D, Yellin J, McKinney R. MD-PhD Program Graduates' Engagement in Research: Results of a National Study. Acad Med. 2021;96(4):540-8.^

^16. Dahn HM, Best L, Bowes D. Attitudes Towards Research During Residency Training: a Survey of Canadian Radiation Oncology Residents and Program Directors. J Cancer Educ. 2020;35(6):1111-8.^

^17. Wang MH, Loewen SK, Giuliani M, Fairchild A, Yee D, Debenham BJ. Motivations, Well-Being, and Career Aspirations of Radiation Oncology Resident Physicians in Canada. J Cancer Educ. 2021;36(5):933-40.^

^18. hematology S, Karamyan o, Harutyunyan L, Ghayamyan M, Khachatryan M, Baloyan E, et al. A nationwide survey of gaps in oncology residency education in Armenia and the potential role of journal clubs. J Clin Oncol. 2025;43(16_suppl):e21024-e.^

^19. Kirkwood MK, Levit LA, Accordino MK, Patel MI, Waterhouse DM, Yabroff R, et al. Does exposure matter? Association between exposure to clinical trial conduct during training and intent to participate in clinical trials post-training among oncology fellows: An ASCO State of Cancer Care in America study. JCO Oncology Practice. 2024;20(10_suppl):69-.^

^20. Farhat K, Obradovic A, Siebert A, Tun HN, Noch EK, Kwan JM. Evaluating factors impacting early career physician-scientists' decisions to continue research careers in the United States of America. BMC Med Educ. 2025;25(1):564.^

^21. Garrison HH, Deschamps AM. NIH research funding and early career physician scientists: continuing challenges in the 21st century. FASEB J. 2014;28(3):1049-58.^

^22. Cammarota A, Siebenhüner AR, Olungu C, Szturz P, Güven DC, Puccini A, et al. Research training, barriers, and career development needs of early-career investigators in oncology: an EORTC survey-based study. ESMO Gastrointestinal Oncology. 2025;9.^

^23. Gerber DE, Khushalani NI, Gao A, Zhang S, Clark JW, Messersmith WA, et al. Protected Time, Resources, and Expectations for Academic Oncology Clinical Research Physicians. J Natl Compr Canc Netw. 2025;23(8):301-6.^
